# Obesity: More Than an Inflammatory, an Infectious Disease?

**DOI:** 10.3389/fimmu.2019.03092

**Published:** 2020-01-14

**Authors:** Paola C. L. Leocádio, Reinaldo B. Oriá, Maria Elena Crespo-Lopez, Jacqueline I. Alvarez-Leite

**Affiliations:** ^1^Laboratório de Aterosclerose e Bioquímica Nutricional, Departamento de Bioquímica e Imunologia, Universidade Federal de Minas Gerais, Belo Horizonte, Brazil; ^2^Departamento de Nutrição, Escola de Enfermagem, Universidade Federal de Minas Gerais, Belo Horizonte, Brazil; ^3^Laboratório de Biologia da Cicatrização, Ontogenia e Nutrição de Tecidos, Faculdade de Medicina, Universidade Federal Do Ceará, Fortaleza, Brazil; ^4^Laboratório de Farmacologia Molecular, Instituto de Ciências Biológicas, Universidade Federal Do Pará, Belém, Brazil

**Keywords:** obesity, microbiota, diet, dysbiosis, inflammation

Much is discussed if obesity or diet components modify the “healthy” microbiota or if microbiota modifications trigger events that culminate in obesity. This association is probably reciprocal, and inflammation has crucial participation on it. We will discuss recent studies showing gut microbiome as an obesogenic factor and the mechanisms linked to the associated of diet, microbiota, and low-grade inflammation.

## Can The Gut Microbiota Regulate Body Weight?

Obesity is a growing epidemy, despite the efforts to contain it. The inflammation generated by the adipocyte hypertrophy and hyperplasia initiates crosstalk between adipocyte and resident macrophage (M2) in white adipose tissue (WAT). Once activated, both adipocyte and activated macrophage (M1) release several adipokines that trigger the infiltration of other immune cells such as neutrophils, CD8+ and CD4+ T cells (1). Tissue-resident innate lymphocytes also play an important role in the homeostasis of WAT and, consequently, in obesity. Although this resident lymphocyte plays regulatory and anti-inflammatory properties in non-obese individuals, obesity promotes changes in the profile of these cells (2). Invariant Natural Killer cells (iNKT) and mucosal-associated invariant T cells (MAIT) are important examples. The frequency of iNKT is reduced in WAT in obesity and is inversely related to the degree of obesity, insulin resistance and fasting blood glucose, suggesting that these cells play a role against metabolic disorders associated with obesity (1, 2). MAIT cells also present reduced frequency and change of phenotype in WAT in obesity, reducing IL-10 synthesis and gamma interferon (IFNγ) and increasing IL-17 production (1, 2) and can play an important role in the progression of inflammation (3).

Adipocytes also produce macrophage colony-stimulating factor (M-CSF-1), causing an increased influx of monocytes from bone marrow-derived precursors and regulating macrophage differentiation and survival (4, 5). The expanded WAT also secrets pro-inflammatory and prothrombotic factors such as interleukin (IL)-1β, IL-6, tumoral necrosis factor (TNF), monocytes and macrophages chemoattractant protein (MCP-1/CCL2), C-reactive protein (CRP), tissue factor and factor VII, plasminogen activator inhibitor type-1 (PAI-1) (6). This pro-inflammatory, prothrombotic environment contributes to the onset of obesity-related complications such as metabolic syndrome, insulin resistance, hypertension, and systemic sterile inflammation.

One of the first studies linking obesity and microbiota was conducted by Ley et al. (7), showing that obesity is associated with a specific microbiota profile. The gut microbiota of healthy individuals is mostly composed of *Firmicutes* (70%) and the *Bacteroidetes* (30%). Other minor phyla are *Actinobacteria, Proteobacteria, Fusobacteria*, and *Verrucomicrobia* (8). The genetically obese ob/ob mice have in their microbiota 50% fewer *Bacterioidetes* and a higher proportion of *Firmicutes* when compared to lean mice. This altered ratio between *Firmicutes* and *Bacteroidetes* (F/B ratio) has also been described in obese individuals (9). Nonetheless, obesity in adulthood is influenced by several factors besides the different profiles of gut microbiota and, until now, studies have not found enough consistency to point out specific obesogenic bacteria (10). However, preclinical studies revealed that the obesogenic microbiota profile could be transmitted from twins discordant for obesity to germ-free (GF) mice. When the fecal microbiota of the obese twin is transplanted to GF mice, the mice eventually become obese, the same occurring with the transplantation of microbiota from the lean twin to GF mice. Moreover, obesity was prevented when mice carrying the obese twin's microbiota were kept in the same cage with mice carrying the lean twin's microbiota (11).

## Since Changes In Microbiota Predispose To Obesity, What Determine The Types of Bacteria That Inhabit The Gut?

The influence of microbiota on obesity development and low-grade inflammation seems to occur even before or immediately after birth. The gut-associated lymphoid tissues (GALT) are formed during embryogenesis and become mature during the microbial colonization, after birth. Bacterial antigens were recognized by the intestinal epithelium via pattern recognition receptors (PRR), such as Toll-like receptors (TLRs) and nucleotide-binding oligomerization domain 1 (NOD-1) (12, 13). Changes in the microbial composition, which occur in the presence of obesity, disrupt the barrier integrity promoted by GALT, increase the intestinal permeability, favor bacterial translocation that triggers the inflammatory process (14).

**Figure 1:**
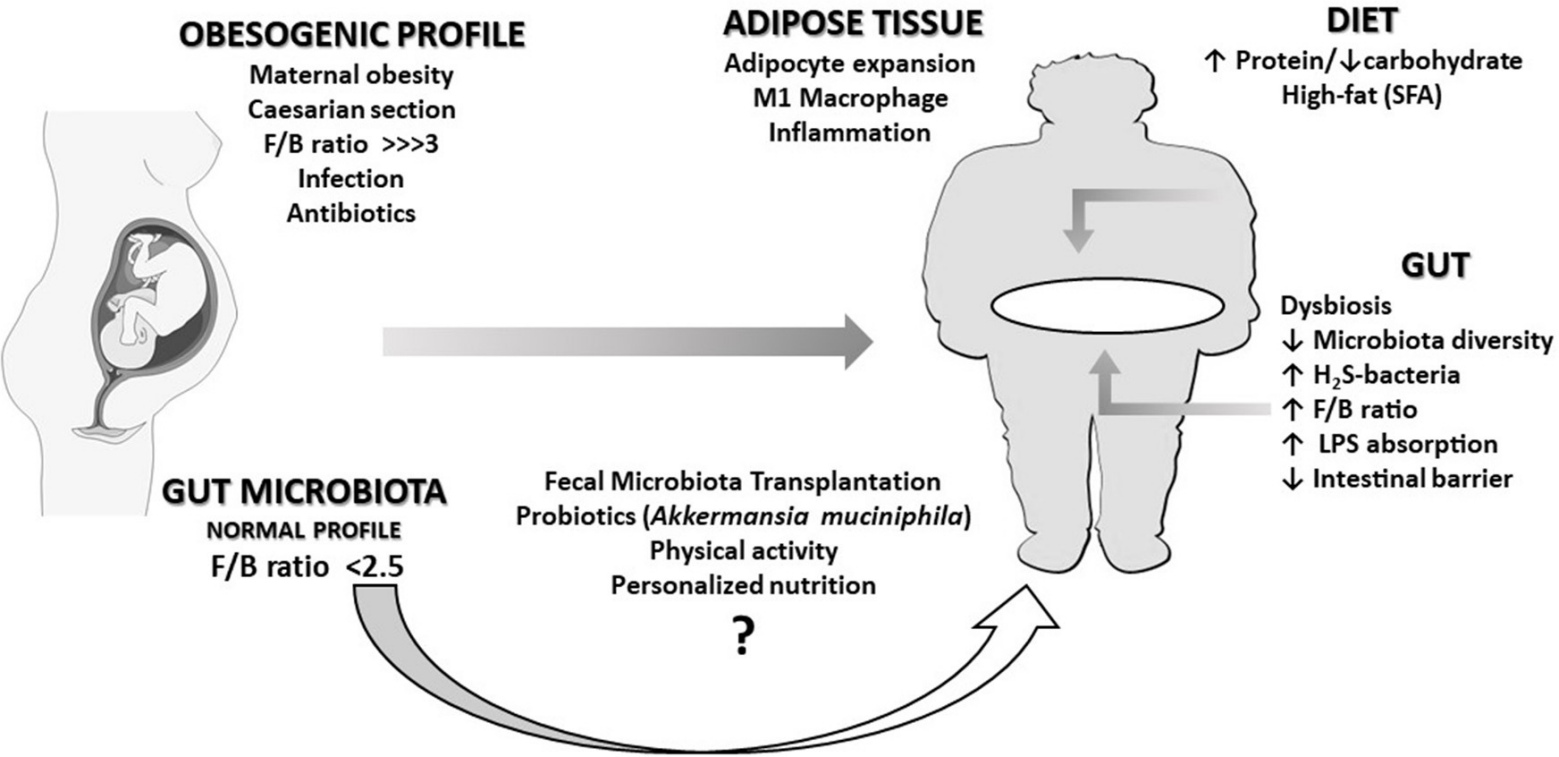
An overview of the relationships described in this opinion paper. An obesogenic profile (characterized by a very high *Firmicutes*/*Bacteroidetes* ratio, F/B) can be caused in the fetus by conditions such as maternal obesity, caesarian section, infections, or antibiotics treatments during pregnancy. The immune and pro-inflammatory response caused by intestinal dysbiosis over life can eventually lead the individual to obesity in adulthood. This scenario can be worsened by the chronic intake of a high-fat diet, responsible for the increase of bacteria producing hydrogen disulfide (H_2_S-bacteria) and pathogenic bacterial lipopolysaccharide (LPS) translocation. A healthy dietary pattern and physical activity may contribute to revert dysbiosis. Although probiotics and fecal microbiota transplantation could eventually improve this condition, presently, there is not enough clinical evidence supporting the adoption of such intervention.

Maternal obesity, caesarian section (CS), infections, and antibiotic utilization were described as factors influencing obesity (15) (Figure 1). Antibiotic therapy in the perinatal period is associated with intestinal microbiota disruption and metabolic changes sufficiently strong to affect body composition in late childhood (16, 17). Indeed, babies from mothers receiving antibiotics during the last gestational trimester presented an 84% higher risk of obesity (16). Moreover, CS is associated with the reduction in *Bacteroidetes* abundance and microbiota diversity in the first 2 years of life. Systemic levels of CXCL10 and CXCL11 chemokines were also reduced in children born by CS (17). Young adults born by CS have a higher risk for increased central and peripheral adiposity than those born by vaginal delivery (18). These associations are stronger in children whose mothers were obese compared to children of non-obese mothers (19).

## What Is The Participation of The Inflammation In This Scenario?

Previous studies clarified the crosstalk between the immune system and microbiota in obesity (20). The IgA is produced by intestinal B cells after interaction with T follicular helper cells (TFH) and secreted into the gut lumen covering bacteria membrane and reducing gut colonization (20, 21). Although bacteria-IgA binding participates in hosting defense against pathogens, IgA can also regulate the gene expression of some gut bacteria population and intestinal cells. It has been proposed that IgA promotes colonization of a healthy microbiota reducing dysbiosis (22). It was tested in MyD88^−/−^ mice that develop obesity faster than controls and are defective in TFH and IgA (23). The expansion of WAT in MyD88^−/−^ is associated with the increase of *Desulfovibrio* and the loss of *Clostridia* populations. When mice were treated with antibiotics or replacement of *Clostridia*, the weight gain was reduced, confirming a cause-effect interaction (20). It suggests that by regulating IgA production, TFH cells maintain the intestinal *Clostridia* population, reducing fatty acids (FA) absorption and protecting the host against obesity.

Previous studies addressed the interaction of microbiota, and pro-inflammatory markers (24) showed that *Bifidobacterium, Faecalibacterium, Ruminococcus*, and *Prevotella* genus abundances were inversely associated with blood levels of CRP or pro-inflammatory cytokines (14, 25–29). Besides the abundance of a specific genus, gut microbial diversity has also been related to obesity. Individuals with low microbial diversity presented higher blood leukocyte count and CRP level that is related to higher triglyceridemia and lower high-density lipoprotein (HDL) levels, insulin resistance and increased risk of atherosclerosis-associated disorders (30).

The decrease in commensal bacteria levels and diversity (dysbiosis) permit the establishment of foreign bacteria, increasing the lipopolysaccharide (LPS) concentration in the gut lumen (Figure 1). LPS can reach systemic circulation by crossing the intestinal mucosa through altered tight junctional complex or linked to dietary fat incorporated into chylomicrons. In the plasma, LPS is transported bound to lipoproteins. Initially, LPS is transported in chylomicrons and then distributed to the other lipoproteins, mainly HDL (31). LPS increases the scavenger receptor binding to lipoproteins, as well as the endocytoses in endothelium and adipocytes. The expanded adipocytes and activated macrophages internalize LPS-rich lipoproteins (32), perpetuating the expansion and inflammation of the WAT. Indeed, LPS triggers the innate immune response on macrophages and adipocytes via TLR4 signaling, resulting in nuclear factor-kappa B (NF-κB) release and pro-inflammatory cytokine production (14, 33).

## How Can The Diet Favor The Obesogenic Microbiota?

Previous studies have demonstrated the effect of high-fat diets (HFD) in increasing *Firmicutes/Bacteroidetes* ratio and in inducing dysbiosis (34–40) (Figure 1). Not only the amount of fat but also the type of FA may influence microbiota. Saturated FA (SFA) promotes dysbiosis by increasing H_2_S-bacteria, which results in the disruption of epithelial integrity by suppression of the tight junction proteins (41). Comparing the effects of HFD with different FAs, SFA quickly and persistently increased the proportion of H_2_S-bacteria over time. When SFA was replaced by ω6-polyunsaturated FAs (ω6-PUFA), the proportion of H_2_S-bacteria remained stable, while replacing SFA for ω3-PUFA, the proportion of H_2_S-bacteria was reduced. This result aggregates beneficial effects to ω3-PUFA, a well-known systemic anti-inflammatory agent.

HFD may also favor obesity not only by promoting dysbiosis but directly by favoring the entry of bacterial components such as LPS (42) (Figure 1). As mentioned before, the absorption of dietary fat facilitates the absorption of LPS since both are transported by chylomicron (43). In the WAT, LPS and palmitic acid increase expression of chemokines and cytokines such as MCP-1 and IL-1β, and inflammation-related enzymes such cyclooxygenase-2, inducing macrophages infiltration and adipocyte expansion. In the liver, palmitic acid also increases the ceramide synthesis of CD36 and free-fatty-acid receptor-1 (FFA1/Gpr40) (41).

Protein-rich/carbohydrate-poor diet may also lead to dysbiosis, changes in barrier integrity and inflammatory activity. Unabsorbed proteins reach the colon, where microbiota exchanges fermentation substrate from carbohydrates to proteins, increasing colonic transit time and pH (41, 44). Protein fermentation increases H_2_S, reactive oxygen species and ammonia production and reduces butyrate and *Roseburia/Eubacterium* abundance, suggesting a worse microbiota profile (45–47). Nonetheless, microbial metabolites from the proteolysis of the essential amino acid tryptophan also influence and modulate host microbiota. Indole groups bind aryl hydrocarbon receptor (AHR) that interfere with several metabolic steps, activate the immune system and reduce intestinal permeability (48).

The presence of non-digested carbohydrates in the colon increases the short-chain FAs produced by microbiota fermentation. These FAs can be absorbed and contribute to the host energy input. In addition to the additional energy absorption caused by short-chain FAs absorption, dysbiosis decreases the expression of FIAF (a lipase lipoprotein inhibitor), stimulating fat deposition in the WAT (33).

## How Are We Fighting Obesity-Related Dysbiosis?

Changing in diet and physical activity are crucial points in the treatment of obesity. Some studies suggest that such changes can alter not only bodyweight but also the microbiota in those individuals. The effects of physical activity modifying microbiota composition and metabolism have been studied, but the results are still controversial (49). Previous studies (50, 51) observed in HFD-fed animals that moderate and high-intensity exercise induced an abundance of *Bacteroidetes* in the colon. Nonetheless, an abundance of *Firmicutes* after physical exercise was also observed in animals with and without diabetes compared to sedentary ones (52). Thus, the influence of exercise on microbiota needs to be carefully evaluated.

Some of the well-established approaches, such as adopting a healthy dietary pattern (53–55), by reducing saturated fat and increasing fiber and antioxidant compounds intake (56, 57) have partially reverse dysbiosis and obesity in experimental studies. Nonetheless, it seems not to be enough to control obesity epidemy. Furthermore, new insights using pre and probiotics and fecal microbiota transplantation (FMT) have now been tested in humans (Figure 1).

*Akkermansia muciniphila*, which is a mucin-degrading bacterium that resides in the mucus layer, has been the most studied, mainly in animal models (58, 59). Clinical studies (60, 61) showed that, in overweight/obese individuals, the oral supplementation of *A. muciniphila* reduced insulin resistance and plasma total cholesterol and levels of blood markers for liver dysfunction and inflammation. However, there was only a modest effect on body weight and composition with *A. muciniphila* supplementation.

Although FMT could be a rational strategy to treat obesity-linked dysbiosis (62), few clinical studies have assessed FMT in individuals with metabolic syndrome or obesity (63–67). Results are until now disappointing, despite the improvement in insulin sensitivity seen in two studies (66, 67), none of them presented promising results in terms of weight loss or reduction in the inflammatory profile. It is confirmed by recent reviews (68, 69) reinforcing the need for studies evaluating the mechanisms by which FMT affect host metabolism and its long-term effects. Moreover, the best preparation, concentration and form of administration of FMT should be defined.

In summary, the study of the complex network formed by gut microbiota, obesity, and inflammation are only in its first steps. The role of the dysbiosis in the genesis of obesity has been progressively uncovered, and the infectious component of this disease has gained more interest. However, up to date, no intervention based on microbes was able to reduce body weight effectively and persistently. Considering the relatively well-established relationship between microbiota and obesity in preclinical studies, additional efforts are necessary for the development of clinical interventions that support the microbiota manipulation as a realistic alternative to combat obesity.

## Author Contributions

PL and JA-L wrote the paper. MC-L and RO revised the paper.

### Conflict of Interest

The authors declare that the research was conducted in the absence of any commercial or financial relationships that could be construed as a potential conflict of interest.

## References

[1] Kane H Lynch L. Innate immune control of adipose tissue homeostasis. Trends Immunol. (2019) 40:857–72. 10.1016/j.it.2019.07.006 31399336

[2] Del Cornò M Conti L Gessani S Innate lymphocytes in adipose tissue homeostasis and their alterations in obesity and colorectal cancer. Front Immunol. (2018) 9:2556 10.3389/fimmu.2018.02556 30455701 PMC6230679

[3] Chehimi M Vidal H Eljaafari A. Pathogenic role of IL-17-producing immune cells in obesity, and related inflammatory diseases. J Clin Med. (2017) 6:68. 10.3390/jcm6070068 28708082 PMC5532576

[4] Weisberg SP McCann D Desai M Rosenbaum M Leibel RL Ferrante AW. Obesity is associated with macrophage accumulation in adipose tissue. J Clin Invest. (2003) 112:1796–808. 10.1172/JCI200319246 14679176 PMC296995

[5] Engin AB. Adipocyte-macrophage cross-talk in obesity. Adv Exp Med Biol. 960:327–43. 10.1007/978-3-319-48382-5_14 28585206

[6] Liu R Nikolajczyk BS. Tissue immune cells fuel obesity-associated inflammation in adipose tissue and beyond. Front Immunol. (2019) 10:1587. 10.3389/fimmu.2019.01587 31379820 PMC6653202

[7] Ley RE Backhed F Turnbaugh P Lozupone CA Knight RD Gordon JI. Obesity alters gut microbial ecology. Proc Natl Acad Sci USA. (2005) 102:11070–5. 10.1073/pnas.0504978102 16033867 PMC1176910

[8] Belizário JE Faintuch J Garay-Malpartida M. Gut microbiome dysbiosis and immunometabolism: new frontiers for treatment of metabolic diseases. Mediat Inflamm. (2018) 2018:1–12. 10.1155/2018/2037838 30622429 PMC6304917

[9] Ley RE Turnbaugh PJ Klein S Gordon JI Human gut microbes associated with obesity Two. Nature. (2006) 444:1022–3. 10.1038/4441022a 17183309

[10] Castaner O Goday A Park YM Lee SH Magkos F Shiow SATE The gut microbiome profile in obesity: a systematic review. Int J Endocrinol. (2018) 2018:1–9. 10.1155/2018/4095789 PMC5933040 29849617

[11] Ridaura VK Faith JJ Rey FE Cheng J Duncan AE Kau AL. Gut microbiota from twins discordant for obesity modulate metabolism in mice. Science. (2013) 341:1241214. 10.1126/science.1241214 24009397 PMC3829625

[12] Renz H Brandtzaeg P Hornef M. The impact of perinatal immune development on mucosal homeostasis and chronic inflammation. Nat Rev Immunol. (2012) 12:9–23. 10.1038/nri3112 22158411

[13] Bouskra D Brézillon C Bérard M Werts C Varona R Boneca IG. Lymphoid tissue genesis induced by commensals through NOD1 regulates intestinal homeostasis. Nature. (2008) 456:507–10. 10.1038/nature07450 18987631

[14] Gomes JMG Costa J de A Alfenas R de CG. Metabolic endotoxemia and diabetes mellitus: a systematic review. Metabolism. (2017) 68:133–44. 10.1016/j.metabol.2016.12.009 28183445

[15] Milani C Duranti S Bottacini F Casey E Turroni F Mahony J. The first microbial colonizers of the human gut: composition, activities, and health implications of the infant gut microbiota. Microbiol Mol Biol Rev. (2017) 81:e00036–17. 10.1128/MMBR.00036-17 29118049 PMC5706746

[16] Mueller NT Whyatt R Hoepner L Oberfield S Dominguez-Bello MG Widen EM. Prenatal exposure to antibiotics, cesarean section and risk of childhood obesity. Int J Obes. (2015) 39:665–70. 10.1038/ijo.2014.180 25298276 PMC4390478

[17] Jakobsson HE Abrahamsson TR Jenmalm MC Harris K Quince C Jernberg C. Decreased gut microbiota diversity, delayed Bacteroidetes colonisation and reduced Th1 responses in infants delivered by Caesarean section. Gut. (2014) 63:559–66. 10.1136/gutjnl-2012-303249 23926244

[18] Mesquita DN Barbieri MA Goldani HAS Cardoso VC Goldani MZ Kac G. Cesarean section is associated with increased peripheral and central adiposity in young adulthood: cohort study. PLoS ONE. (2013) 8:e66827. 10.1371/journal.pone.0066827 23826150 PMC3694972

[19] Blustein J Attina T Liu M Ryan AM Cox LM Blaser MJ. Association of caesarean delivery with child adiposity from age 6 weeks to 15 years. Int J Obes. (2013) 37:900–6. 10.1038/ijo.2013.49 23670220 PMC5007946

[20] Petersen C Bell R Klag KA Lee S-H Soto R Ghazaryan A. T cell–mediated regulation of the microbiota protects against obesity. Science. (2019) 365:eaat9351. 10.1126/science.aat9351 31346040 PMC7294966

[21] Wang Y Hooper L V. Immune control of the microbiota prevents obesity. Science. (2019) 365:316–17. 10.1126/science.aay2057 31346050

[22] Donaldson GP Ladinsky MS Yu KB Sanders JG Yoo BB Chou WC. Gut microbiota utilize immunoglobulin a for mucosal colonization. Science. (2018) 360:795–800. 10.1126/science.aaq0926 29724905 PMC5973787

[23] Kubinak JL Petersen C Stephens WZ Soto R Bake E O'Connell RM. MyD88 signaling in T cells directs IgA-mediated control of the microbiota to promote health. Cell Host Microbe. (2015) 17:153–63. 10.1016/j.chom.2014.12.009 25620548 PMC4451207

[24] van den Munckhof ICL Kurilshikov A ter Horst R Riksen NP Joosten LAB Zhernakova A. Role of gut microbiota in chronic low-grade inflammation as potential driver for atherosclerotic cardiovascular disease: a systematic review of human studies. Obes Rev. (2018) 19:1719–34. 10.1111/obr.12750 30144260

[25] Le Chatelier E Nielsen T Qin J Prifti E Hildebrand F Falony G. Richness of human gut microbiome correlates with metabolic markers. Nature. (2013) 500:541–6. 10.1038/nature12506 23985870

[26] Cândido FG Valente FXFX Grześkowiak ŁM Moreira APB Rocha DMUP Alfenas RCG. Impact of dietary fat on gut microbiota and low-grade systemic inflammation: mechanisms and clinical implications on obesity. Int J Food Sci Nutr. (2018) 69:125–43. 10.1080/09637486.2017.1343286 28675945

[27] Furet J-P Kong L-C Tap J Poitou C Basdevant A Bouillot J-L. Differential adaptation of human gut microbiota to bariatric surgery–induced weight loss. Diabetes. (2010) 59:3049–57. 10.2337/db10-0253 20876719 PMC2992765

[28] Rajkumar H Mahmood N Kumar M Varikuti SR Challa HR Myakala SP Effect of probiotic (VSL#3) and omega-3 on lipid profile, insulin sensitivity, inflammatory markers, and gut colonization in overweight adults: a randomized, controlled trial. Mediat Inflamm. (2014) 2014:348959 10.1155/2014/348959 PMC3984795 24795503

[29] Martínez I Lattimer JM Hubach KL Case JA Yang J Weber CG. Gut microbiome composition is linked to whole grain-induced immunological improvements. ISME J. (2013) 7:269–80. 10.1038/ismej.2012.104 23038174 PMC3554403

[30] Manco M Putignani L Bottazzo GF. Gut microbiota, lipopolysaccharides, and innate immunity in the pathogenesis of obesity and cardiovascular risk. Endocr Rev. (2010) 31:817–44. 10.1210/er.2009-0030 20592272

[31] Hersoug LG Møller P Loft S. Role of microbiota-derived lipopolysaccharide in adipose tissue inflammation, adipocyte size and pyroptosis during obesity. Nutr Res Rev. (2018) 31:153–63. 10.1017/S0954422417000269 29362018

[32] Hersoug L-G Møller P Loft S. Gut microbiota-derived lipopolysaccharide uptake and trafficking to adipose tissue: implications for inflammation and obesity. Obes Rev. (2016) 17:297–312. 10.1111/obr.12370 26712364

[33] Muscogiuri G Cantone E Cassarano S Tuccinardi D Barrea L Savastano S. Gut microbiota: a new path to treat obesity. Int J Obes Suppl. (2019) 9:10–19. 10.1038/s41367-019-0011-7 31391921 PMC6683132

[34] Turnbaugh PJ Ley RE Mahowald MA Magrini V Mardis ER Gordon JI. An obesity-associated gut microbiome with increased capacity for energy harvest. Nature. (2006) 444:1027–31. 10.1038/nature05414 17183312

[35] Cani PD Bibiloni R Knauf C Waget A Neyrinck AM Delzenne NM. Changes in gut microbiota control metabolic endotoxemia-induced inflammation in high-fat diet-induced obesity and diabetes in mice. Diabetes. (2008) 57:1470–81. 10.2337/db07-1403 18305141

[36] Ding S Chi MM Scull BP Rigby R Schwerbrock NMJ Magness S. High-fat diet: bacteria interactions promote intestinal inflammation which precedes and correlates with obesity and insulin resistance in mouse. PLoS ONE. (2010) 5:e12191. 10.1371/journal.pone.0012191 20808947 PMC2922379

[37] Nguyen SG Kim J Guevarra RB Lee JH Kim E Kim SI. Laminarin favorably modulates gut microbiota in mice fed a high-fat diet. Food Funct. (2016) 7:4193–201. 10.1039/C6FO00929H 27713958

[38] Turnbaugh PJ Hamady M Yatsunenko T Cantarel BL Duncan A Ley RE. A core gut microbiome in obese and lean twins. Nature. (2009) 457:480–4. 10.1038/nature07540 19043404 PMC2677729

[39] Araujo JR Tomas J Brenner C Sansonetti PJ Araújo JR Tomas J. Impact of high-fat diet on the intestinal microbiota and small intestinal physiology before and after the onset of obesity. Biochimie. (2017) 141:97–106. 10.1016/j.biochi.2017.05.019 28571979

[40] Ussar S Griffin NW Bezy O Fujisaka S Vienberg S Softic S. Interactions between gut microbiota, host genetics and diet modulate the predisposition to obesity and metabolic syndrome. Cell Metab. (2015) 22:516–30. 10.1016/j.cmet.2015.07.007 26299453 PMC4570502

[41] Wisniewski PJ Dowden RA Campbell SC. Role of dietary lipids in modulating inflammation through the gut microbiota. Nutrients. (2019) 11:E117. 10.3390/nu11010117 30626117 PMC6357048

[42] Burcelin R Garidou L Pomié C. Immuno-microbiota cross and talk: the new paradigm of metabolic diseases. Semin Immunol. (2012) 24:67–74. 10.1016/j.smim.2011.11.011 22265028

[43] Erridge C Attina T Spickett CM Webb DJ. A high-fat meal induces low-grade endotoxemia: evidence of a novel mechanism of postprandial inflammation. Am J Clin Nutr. (2007) 86:1286–92. 10.1093/ajcn/86.5.1286 17991637

[44] Roager HM Hansen LBS Bahl MI Frandsen HL Carvalho V Gøbel RJ. Colonic transit time is related to bacterial metabolism and mucosal turnover in the gut. Nat Microbiol. (2016) 1:16093. 10.1038/nmicrobiol.2016.93 27562254

[45] Geypens B Claus D Evenepoel P Hiele M Maes B Peeters M. Influence of dietary protein supplements on the formation of bacterial metabolites in the colon. Gut. (1997) 41:70–6. 10.1136/gut.41.1.70 9274475 PMC1027231

[46] Nyangale EP Mottram DS Gibson GR. Gut microbial activity, implications for health and disease: the potential role of metabolite analysis. J Proteome Res. (2012) 11:5573–85. 10.1021/pr300637d 23116228

[47] Russell WR Gratz SW Duncan SH Holtrop G Ince J Scobbie L. High-protein, reduced-carbohydrate weight-loss diets promote metabolite profiles likely to be detrimental to colonic health. Am J Clin Nutr. (2011) 93:1062–72. 10.3945/ajcn.110.002188 21389180

[48] Roager HM Licht TR. Microbial tryptophan catabolites in health and disease. Nat Commun. (2018) 9:3294. 10.1038/s41467-018-05470-4 30120222 PMC6098093

[49] Bianchi F Duque ALRF Saad SMI Sivieri K. Gut microbiome approaches to treat obesity in humans. Appl Microbiol Biotechnol. (2019) 103:1081–94. 10.1007/s00253-018-9570-8 30554391

[50] Evans CC LePard KJ Kwak JW Stancukas MC Laskowski S Dougherty J. Exercise prevents weight gain and alters the gut microbiota in a mouse model of high fat diet-induced obesity. PLoS ONE. (2014) 9:e92193. 10.1371/journal.pone.0092193 24670791 PMC3966766

[51] Denou E Marcinko K Surette MG Steinberg GR Schertzer JD. High-intensity exercise training increases the diversity and metabolic capacity of the mouse distal gut microbiota during diet-induced obesity. Am J Physiol Endocrinol Metab. (2016) 310:E982–93. 10.1152/ajpendo.00537.2015 27117007 PMC4935139

[52] Lambert JE Myslicki JP Bomhof MR Belke DD Shearer J Reimer RA. Exercise training modifies gut microbiota in normal and diabetic mice. Appl Physiol Nutr Metab. (2015) 40:749–52. 10.1139/apnm-2014-0452 25962839

[53] Haro C Garcia-Carpintero S Alcala-Diaz JF Gomez-Delgado F Delgado-Lista J Perez-Martinez P. The gut microbial community in metabolic syndrome patients is modified by diet. J Nutr Biochem. (2016) 27:27–31. 10.1016/j.jnutbio.2015.08.011 26376027

[54] Lopez-Legarrea P Fuller NR Zulet MA Martinez JA Caterson ID. The influence of Mediterranean, carbohydrate and high protein diets on gut microbiota composition in the treatment of obesity and associated inflammatory state. Asia Pac J Clin Nutr. (2014) 23:360–8. 10.6133/apjcn.2014.23.3.16 25164445

[55] Del Chierico F Vernocchi P Dallapiccola B Putignani L. Mediterranean diet and health: food effects on gut microbiota and disease control. Int J Mol Sci. (2014) 15:11678. 10.3390/ijms150711678 24987952 PMC4139807

[56] Pandey KB Rizvi SI. Plant polyphenols as dietary antioxidants in human health and disease. Oxid Med Cell Longev. (2009) 2:270–8. 10.4161/oxim.2.5.9498 20716914 PMC2835915

[57] Roopchand DE Carmody RN Kuhn P Moskal K Rojas-Silva P Turnbaugh PJ. Dietary polyphenols promote growth of the gut bacterium *Akkermansia muciniphila* and attenuate high-fat diet–induced metabolic syndrome. Diabetes. (2015) 64:2847–58. 10.2337/db14-1916 25845659 PMC4512228

[58] Chelakkot C Choi Y Kim DK Park HT Ghim J Kwon Y. *Akkermansia muciniphila*-derived extracellular vesicles influence gut permeability through the regulation of tight junctions. Exp Mol Med. (2018) 50:e450–11. 10.1038/emm.2017.282 29472701 PMC5903829

[59] Li J Lin S Vanhoutte PM Woo CW Xu A *Akkermansia muciniphila* protects against atherosclerosis by preventing metabolic endotoxemia-induced inflammation in apoe^−/−^ mice. Circulation. (2016) 133:2434–46. 10.1161/CIRCULATIONAHA.115.019645 27143680

[60] Depommier C Everard A Druart C Plovier H Van Hul M Vieira-Silva S. Supplementation with *Akkermansia muciniphila* in overweight and obese human volunteers: a proof-of-concept exploratory study. (2019). 25:1. 10.1038/s41591-019-0495-2 31263284 PMC6699990

[61] Dao MC Everard A Aron-Wisnewsky J Sokolovska N Prifti E Verger EO. *Akkermansia muciniphila* and improved metabolic health during a dietary intervention in obesity: relationship with gut microbiome richness and ecology. Gut. (2016) 65:426–36. 10.1136/gutjnl-2014-308778 26100928

[62] Lee P Yacyshyn BR Yacyshyn MB. Gut microbiota and obesity: an opportunity to alter obesity through faecal microbiota transplant (FMT). Diabetes, Obes Metab. (2019) 21:479–90. 10.1111/dom.13561 30328245

[63] Allegretti JR Kassam Z Mullish BH Chiang A Carrellas M Hurtado J. Effects of fecal microbiota transplantation with oral capsules in obese patients. Clin Gastroenterol Hepatol. (2019) 10.1016/j.cgh.2019.07.006. [Epub ahead of print]. 31301451

[64] Allegretti JR Mullish BH Kelly C Fischer M. The evolution of the use of faecal microbiota transplantation and emerging therapeutic indications. Lancet. (2019) 394:420–31. 10.1016/S0140-6736(19)31266-8 31379333

[65] Smits LP Kootte RS Levin E Prodan A Fuentes S Zoetendal EG. Effect of vegan fecal microbiota transplantation on carnitine- and choline-derived trimethylamine-N-oxide production and vascular inflammation in patients with metabolic syndrome. J Am Heart Assoc. (2018) 7:e008342. 10.1161/JAHA.117.008342 29581220 PMC5907601

[66] Vrieze A Van Nood E Holleman F Salojärvi J Kootte RS Bartelsman JFWM. Transfer of intestinal microbiota from lean donors increases insulin sensitivity in individuals with metabolic syndrome. Gastroenterology. (2012) 143:913–16.e7. 10.1053/j.gastro.2012.06.031 22728514

[67] Kootte RS Levin E Salojärvi J Smits LP Hartstra AV Udayappan SD. Improvement of insulin sensitivity after lean donor feces in metabolic syndrome is driven by baseline intestinal microbiota composition. Cell Metab. (2017) 26:611–19.e6. 10.1016/j.cmet.2017.09.008 28978426

[68] Aron-Wisnewsky J Prifti E Belda E Ichou F Kayser BD Dao MC. Major microbiota dysbiosis in severe obesity: fate after bariatric surgery. Gut. (2019) 68:70–82. 10.1136/gutjnl-2018-316103 29899081 PMC7143256

[69] Zhang Z Mocanu V Cai C Dang J Slater L Deehan EC. Impact of fecal microbiota transplantation on obesity and metabolic syndrome—a systematic review. Nutrients. (2019) 11:2291. 10.3390/nu11102291 31557953 PMC6835402

